# Prognostic utility and characterization of cell-free DNA in patients with severe sepsis

**DOI:** 10.1186/cc11466

**Published:** 2012-08-13

**Authors:** Dhruva J Dwivedi, Lisa J Toltl, Laura L Swystun, Janice Pogue, Kao-Lee Liaw, Jeffrey I Weitz, Deborah J Cook, Alison E Fox-Robichaud, Patricia C Liaw

**Affiliations:** 1 Department of Medicine, McMaster University, 1280 Main St. W., Hamilton, ONT, L8S 4K1, Canada; 2 Department of Medical Sciences, McMaster University, 1280 Main St. W., Hamilton, ONT, L8S 4K1, Canada; 3 Department of Clinical Epidemiology and Biostatistics, McMaster University, 1280 Main St. W., Hamilton, ONT, L8S 4K1, Canada; 4 Population Health Research Institute, Hamilton Health Sciences and McMaster University, 237 Barton St. E., Hamilton, ONT, L8L 2X2, Canada; 5 Department of Geography and Earth Sciences, 1280 Main St. W., Hamilton, ONT, L8S 4K1, Canada; 6 St. Joseph's Healthcare Hamilton, 50 Charlton Ave. E, Hamilton, ONT, L8N 4A6, Canada; 7 Thrombosis and Atherosclerosis Research Institute (TaARI), 237 Barton St. E., Hamilton, ONT, L8L 2X2, Canada

## Abstract

**Introduction:**

Although sepsis is the leading cause of death in noncoronary critically ill patients, identification of patients at high risk of death remains a challenge. In this study, we examined the incremental usefulness of adding multiple biomarkers to clinical scoring systems for predicting intensive care unit (ICU) mortality in patients with severe sepsis.

**Methods:**

This retrospective observational study used stored plasma samples obtained from 80 severe sepsis patients recruited at three tertiary hospital ICUs in Hamilton, Ontario, Canada. Clinical data and plasma samples were obtained at study inclusion for all 80 patients, and then daily for 1 week, and weekly thereafter for a subset of 50 patients. Plasma levels of cell-free DNA (cfDNA), interleukin 6 (IL-6), thrombin, and protein C were measured and compared with clinical characteristics, including the primary outcome of ICU mortality and morbidity measured with the Multiple Organ Dysfunction (MODS) score and Acute Physiology and Chronic Health Evaluation (APACHE) II scores.

**Results:**

The level of cfDNA in plasma at study inclusion had better prognostic utility than did MODS or APACHE II scores, or the biomarkers measured. The area under the receiver operating characteristic (ROC) curves for cfDNA to predict ICU mortality is 0.97 (95% CI, 0.93 to 1.00) and to predict hospital mortality is 0.84 (95% CI, 0.75 to 0.94). We found that a cfDNA cutoff value of 2.35 ng/μl had a sensitivity of 87.9% and specificity of 93.5% for predicting ICU mortality. Sequential measurements of cfDNA suggested that ICU mortality may be predicted within 24 hours of study inclusion, and that the predictive power of cfDNA may be enhanced by combining it with protein C levels or MODS scores. DNA-sequence analyses and studies with Toll-like receptor 9 (TLR9) reporter cells suggests that the cfDNA from sepsis patients is host derived.

**Conclusions:**

These studies suggest that cfDNA provides high prognostic accuracy in patients with severe sepsis. The serial data suggest that the combination of cfDNA with protein C and MODS scores may yield even stronger predictive power. Incorporation of cfDNA in sepsis risk-stratification systems may be valuable for clinical decision making or for inclusion into sepsis trials.

## Introduction

Sepsis is a devastating condition characterized by systemic activation of inflammatory and coagulation pathways in response to microbial infection of normally sterile parts of the body [1,2]. Microbial invasion originates from a breach of integrity of the host barrier, either physical or immunologic. Sepsis is the leading cause of death in critically ill patients and is a leading cause of morbidity and mortality in the Western world [3]. Severe sepsis, defined as sepsis associated with at least one dysfunctional organ, afflicts approximately 750,000 people in the United States annually, with an estimated mortality rate of 30% to 50% [3].

The identification of highly reliable outcome predictors in severe sepsis is important to describe disease severity for bedside prognosis, to assist deciding on location of care, to monitor response to treatment, and to stratify or enroll patients in clinical trials. However, the heterogeneity of patients with severe sepsis makes the identification of those at high risk of death a challenge, both for clinical and research purposes.

Various clinical scoring systems have been developed to facilitate evaluation of disease severity, each with its own limitations [4]. These scoring systems can be divided into two main classes. The first class of scoring system assesses disease severity primarily through evaluation of physiological parameters (for example, Acute Physiology and Chronic Health Evaluation [APACHE] II score [5]). These scores are somewhat laborious to use and are primarily considered at the time of admission to the intensive care unit (ICU). In contrast, organ-dysfunction scores can more readily be measured over time and thus capture the dynamics of organ dysfunction, including the patients' response to therapeutic interventions. Examples are the Multiple Organ Dysfunction Scores [MODS] [6] and Sequential Organ Failure Assessment [SOFA] [7] scores. However, both of these classes of scoring systems focus only on physiological abnormalities, and they are not exclusive to patients with sepsis syndrome. Furthermore, these scores have only a moderate discriminative power with respect to ICU mortality. With receiver operating characteristic (ROC) curves [8], which measure the diagnostic accuracy of a given test, the area under the curve (AUC) for these scores ranges from 0.6 to 0.7 [9].

Several biomarkers have been proposed to be of potential use for sepsis prognostication, including inflammatory cytokines, cell-surface markers, acute-phase proteins, coagulation factors, and apoptosis mediators [10,11]. Recent research suggested that cell-free DNA (cfDNA), released as a result of cell necrosis or of apoptosis, may have prognostic utility in a range of conditions, including cancer [12], trauma [13], stroke [14], myocardial infarction [15], and sepsis [16,17]. However, sample sizes in these biomarker studies remain small, and it is unclear whether any one marker could predict outcome in a complicated condition such as sepsis.

The objective of this study was to examine the incremental usefulness of adding multiple biomarkers to clinical scoring systems for predicting ICU mortality in patients with severe sepsis. We examined the following variables in 80 patients with severe sepsis: age, acute physiology and chronic health evaluation (APACHE II) scores, multiple organ-dysfunction scores (MODS), IL-6 (a proinflammatory cytokine), thrombin (a procoagulant factor), protein C (an anticoagulant factor), and cfDNA. We chose these biomarkers to capture a broad spectrum of physiologic derangements that may be important in sepsis pathophysiology [18-20]. Herein, we report the prognostic value of cfDNA in a cohort of patients with severe sepsis. We also characterized the biochemical properties of cfDNA in sepsis patients.

## Materials and methods

### Patients and plasma samples

Frozen plasma samples were obtained from an available biobank of 80 severe sepsis patients. The patients were recruited between March 2001 and September 2007 from three academic, tertiary care ICUs in Hamilton, Ontario. Patients with severe sepsis were identified by using the inclusion and exclusion criteria as described by Bernard *et al*. [21]. The management of the patients was left to the attending ICU team, following published practice during that time period. Patients who received recombinant Activated Protein C (rAPC) therapy were excluded from the study. This study was approved by the Research Ethics Board of McMaster University and the Hamilton Health Sciences, Hamilton, Ontario, Canada (REB approval 01-195). Written informed consent was obtained from the patient (or substitute decision-maker) or from the healthy controls before blood collection.

The patient blood samples were collected within 24 hours of meeting the inclusion criteria for severe sepsis in the ICU. The blood was processed within 2 hours of blood collection. In brief, venous blood (9 ml total) was collected from indwelling catheters and transferred into 15-ml polypropylene tubes containing 0.5 ml of 0.105 M buffered trisodium citrate (pH 5.4). The blood was centrifuged at 1,500 *g *for 10 minutes at 20°C, and the plasma was stored as 200-μl aliquots at -80°C and thawed at the time of assays.

### Plasma samples from healthy controls

Plasma samples were obtained from 14 healthy adult volunteers who were not receiving any medication at the time of blood sampling. No attempt to match cases and controls was made.

### Quantification of cfDNA levels in plasma

cfDNA was isolated from 250 μl of plasma by using the QIAamp DNA Blood Mini Kit (Qiagen, Valencia, CA, USA). The concentration of the DNA was measured with UV absorbance at 260 nm by using a spectrophotometer (Beckman DU 7400; Beckman Coulter Inc., Brea, CA, USA). The purity of the DNA was confirmed by determining the OD_260_/OD_280 _ratio and by the BCA Protein Assay (Pierce, Rockford, IL, USA).

### Measurement of IL-6, thrombin-antithrombin (TAT) complexes, and protein C in sepsis plasma

IL-6 levels in plasma samples were determined by using the Human IL-6 ELISA Development kit (Biovision, Mountain View, CA, USA). Levels of TAT complexes were quantified by the Enzygnost TAT micro kit (Dade Behring Inc., Marburg, Germany). Total protein C antigen in citrated plasma samples was quantified with an enzyme immunoassay (Affinity Biologicals Inc., Ancaster, ON, Canada).

### Stimulation of Toll-like receptor 9 (TLR-9) reporter cells with cfDNA

To distinguish bacterial DNA from mammalian DNA, we used 293XL-hTLR9 cells, a human TLR9 reporter cell line (InvivoGen, San Diego, CA, USA). To monitor the translocation of nuclear factor κB (NF-κB; which is induced by TLR-9 signaling), we used the pNiFty2 plasmid, which encodes a reporter gene encoding secreted alkaline phosphatase (SEAP). Expression of SEAP is controlled by an NF-κB-inducible promoter. The positive control was ODN 2006, a synthetic oligonucleotide that contains unmethylated CpG dinucleotides in particular sequence contexts (InvivoGen).

### DNA sequence analyses of cfDNA from severe sepsis patients

The prominent DNA bands from the severe sepsis patients (that is, the ~150-bp and ~300-bp DNA bands) were excised from 1.5% agarose gels, blunt ended, inserted into the cloning vector (Litmus 28i), and sequenced.

### Statistical analyses

The LOGISTIC procedure of SAS (version 9.2) was used to assess the dependency of the probability of dying on each predictive variable (either clinical score or biomarker) or on a combination of several variables. By using the variables created by the OUTROC option of the MODEL statement in the LOGISTIC procedure [22], the ROC curves were drawn by using Excel, and the AUCs and their standard errors were computed by using SAS codes, based on the distribution-free formulas provided by Hanley and McNeil [8]. In constructing the 95% confidence intervals for AUC, the critical value (1.96), based on the assumption of the standard normal distribution, is replaced by the critical value based on a *t*-distribution with (n-p-1) degrees of freedom, where n is the sample size, and p is number of explanatory variable(s) used in the logistic model. This replacement has the advantage of allowing the confidence interval to be somewhat wider for the AUC that is generated by more than one explanatory variable. The reason for this replacement is that the use of more explanatory variables results in a reduction in degrees of freedom, which in turn leads to a reduction in confidence.

For temporal changes in biomarkers, analysis of variance (ANOVA) was used to compare the results between survivors and nonsurvivors. For temporal changes in biomarkers, SigmaPlot graphs were used to distinguish the patterns between survivors and nonsurvivors. For each biomarker, the statistical significance of the gap between these two groups of patients was assessed by analysis of variance (ANOVA).

## Results

### Prognostic utility of cfDNA in severe sepsis patients

The baseline (day 1) characteristics of the 80 severe sepsis patients are shown in Table 1 ("baseline" is defined as within 24 hours of meeting the inclusion criteria for severe sepsis in the ICU). We found that the mean baseline level of cfDNA in survivors (1.16 ± 0.13 ng/μl; *n *= 46) was similar to that in healthy volunteers (0.93 ± 0.76 ng/μl; *n *= 14) (*P *= 0.426), whereas the mean cfDNA levels in nonsurvivors are markedly higher (4.65 ± 0.48 ng/μl; *n *= 34) compared with survivors (*P *< 0.001) and with healthy volunteers (*P *< 0.001). The mortality rates increased across increasing quartiles of baseline plasma cfDNA levels (5% in the first quartile, 60% in the second quartile, 83% in the third quartile, and 100% in the fourth quartile). In contrast, the mortality rates in the highest quartiles of baseline APACHE II score, MODS score, and age were 36%, 64%, and 50%, respectively.

**Table 1:** Baseline characteristics of 80 patients with severe sepsis

Characteristic	Survivors *n *= 46	Nonsurvivors *n *= 34
Age, years		
Mean ± SEM (min, max)	60 ± 2.2 (20, 84)	68 ± 2.3 (37, 87)
Gender, % female (no./total)	30.4 (14 of 46)	32.3 (11 of 34)
APACHE II score		
Mean ± SEM (min, max)	21.6 ± 1.2 (7, 40)	24.5 ± 1.1 (10, 34)
MODS score		
Mean ± SEM (min, max)	8.3 ± 0.56 (2, 17)	10.4 ± 0.62 (3, 17)
Primary site of infection, no. (% of total)		
Lung	24 (52.1%)	18 (52.9%)
Blood	11 (23.9%)	4 (11.8%)
Urinary tract	1 (2.2%)	3 (8.8%)
Abdomen	5 (10.9%)	1 (2.9%)
Skin	2 (4.3%)	0 (0%)
Other	1 (2.2%)	5 (14.7%)
Unknown	2 (4.3%)	3 (8.8%)
Positive cultures, no. (% of total)		
Gram-negative bacteria	8 (17.4%)	3 (8.8%)
Gram-positive bacteria	16 (34.8%)	9 (26.5%)
Fungus	2 (4.3%)	4 (11.8%)
Mixed	11 (23.9%)	14 (41.2%)
Unknown	9 (19.6%)	4 (11.8%)

Applying a binomial logistic model to the baseline data, we generated receiver operating characteristic (ROC) curves to determine the predictive power of each clinical score and biomarker for ICU mortality. The area under the curve (AUC) for each ROC curve is summarized in Table 2. With an AUC value of 0.97 (95% CI, 0.93 to 1.00), cfDNA has the strongest predictive power among the eight predictors listed in Table 2. In this cohort of patients, MODS score, APACHE II score, and age have modest predictive powers, with AUC values of 0.63 (95% CI, 0.50 to 0.75), 0.64 (95% CI, 0.52 to 0.77), and 0.66 (95% CI, 0.54 to 0.78), respectively. Thrombin-antithrombin complexes (TAT), protein C, IL-6, and gender have no statistically significant predictive power because the AUC of each of them has a 95% confidence interval that spans the AUC for a completely random process: 0.5.

**Table 2:** ROC analysis to predict ICU mortality in a cohort of 80 severe sepsis patients

Predictor	Area under the curve	95% Confidence interval
cfDNA	0.97	0.93 to 1.00
APACHE II	0.64	0.52 to 0.77
MODS	0.63	0.50 to 0.75
Age	0.66	0.54 to 0.78
TAT complexes	0.57	0.44 to 0.70
Protein C	0.57	0.43 to 0.70
IL-6	0.56	0.43 to 0.69
Female gender	0.51	0.39 to 0.65

ROC curves were generated to determine the predictive power of each variable (baseline values) for ICU mortality. The AUCs for each ROC curve are summarized here.

**Figure 1:**
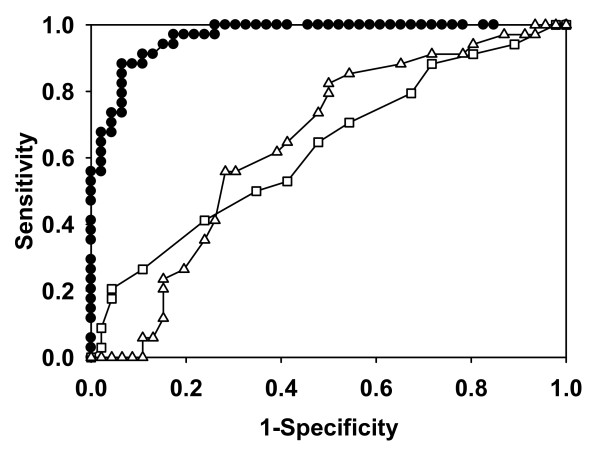
**Receiver operating characteristic (ROC) curves for cfDNA (∙), MOD score (□), and APACHE II score (Δ) to predict ICU mortality in 80 patients with severe sepsis**. **By u**sing a binomial logistic model, we generated ROC curves to determine the predictive power of baseline values of cfDNA, MODS score, and APACHEII score for ICU mortality. The area under the curve (AUC) for cfDNA is 0.97 (95% CI, 0.93 to 1.00), for APACHE II score, is 0.64 (95% CI, 0.52 to 0.77), and for MODS score, is 0.63 (95% CI, 0.50 to 0.75).

The ROC curves for cfDNA, MODS, and APACHE II are displayed in Figure 1. For cfDNA, the best cutoff value that maximizes the sum of sensitivity and specificity is 2.35 ng/μl, a value that yields a sensitivity of 87.9% (95% CI, 77.2 to 99.2) and specificity of 93.5% (95% CI, 86.2 to 100) for predicting ICU mortality. By using the baseline data, we also found that combining cfDNA with any of the other seven predictors does not contribute to any statistically significant improvement in the model's predictive power beyond that achieved by cfDNA alone (data not shown). With respect to hospital mortality, the AUC of cfDNA is 0.84 (95% CI, 0.75 to 0.94), which is higher than the corresponding AUCs of all other predictors.

We also calculated the positive predictive values (PPVs) and negative predictive values (NPVs) for cfDNA and other variables (APACHE II, MODS, age, TAT complexes, protein C, IL-6, gender). As shown in Table 3, the prognostic utility of baseline levels of cfDNA is indicated by a PPV = 0.91 (95% CI, 0.81 to 1.00) and NPV = 0.91 (95% CI, 0.83 to 1.00), which are greater than the corresponding PPVs and NPVs of the other variables.

**Table 3:** Positive predictive value (PPV) and negative predictive value (NPV) for assessing the prognostic utility of the baseline values of cfDNA and other variables

Variable	Positive predictive value	95% CI	Likelihood ratio χ^2^	Negative predictive value	95% CI	Likelihood ratio χ^2^
cfDNA	0.91	0.81-1.00	25.64	0.91	0.83-1.00	37.80
APACHE II	0.55	0.42-0.69	0.64	0.88	0.74-1.00	15.19
MODS	0.65	0.45-0.85	2.16	0.67	0.54-0.79	6.46
Age	0.53	0.39-0.68	0.19	0.73	0.57-0.88	7.07
TAT complexes	0.59	0.40-0.77	0.87	0.65	0.52-0.79	4.67
Protein C	0.64	0.43-0.84	1.66	0.66	0.53-0.78	5.68
IL-6	0.86	0.59-1.00	3.96	0.61	0.50-0.73	3.59
Female gender	0.44	0.30-0.57	0.89	0.60	0.40-0.80	1.01

### Temporal changes in cfDNA, protein C, and MODS in severe sepsis patients

**Figure 2:**
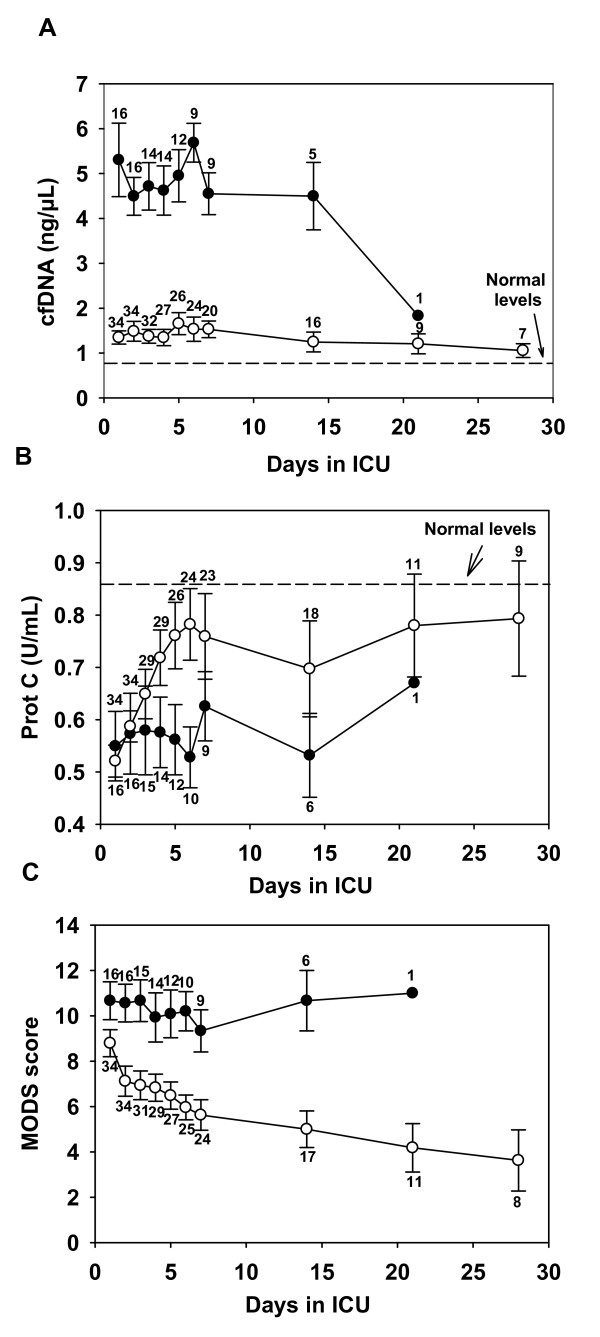
**Temporal changes in levels of cfDNA (A), PC (B), and MOD score (C) in 50 patients with severe sepsis**. Survivors are shown by white circles (o), and nonsurvivors are shown by black circles (●). The number of patients at each time point (for which cfDNA, PC, or MODS values are available) is indicated in each graph. Data are shown as the mean ± SEM. The mean levels of cfDNA and PC in healthy volunteers (*n *= 14) is shown by the arrows.

In the 80 severe sepsis patients, serial plasma samples and MOD scores were available in 50 of the patients (16 survivors, 34 non-survivors). These serial samples and MODS scores were collected in the ICU on days 1 through 7, and then on days 14, 21, and 28. Figure 2A summarizes the temporal changes in cfDNA in these patients. With ANOVA, we found a highly significant difference between cfDNA levels in survivors and nonsurvivors over the course of 28 days (*t *= 19.61). Furthermore, levels of cfDNA at day 1 did not differ significantly from the levels measured in subsequent days. In other words, cfDNA levels in nonsurvivors were high at baseline and remained high; cfDNA levels in survivors were low and remained low.

Abnormalities in coagulation parameters are routinely observed in patients with severe sepsis [23-25]. In this study, we also examined temporal changes in the anticoagulant factor protein C in plasma from severe sepsis patients (Figure 2B). Relative to healthy volunteers, all sepsis patients had reduced levels of protein C at day 1, although no statistically significant difference in protein C levels was found between survivors and nonsurvivors. However, patients who survived exhibited an early return of protein C levels to near-normal levels (*P *< 0.01 when comparing day-1 with day-6 levels of protein C in survivors). In contrast, patients who died exhibited no increase in protein C levels during the course of their ICU stay. The overall difference in protein C level between the survivors and nonsurvivors is significantly different from zero (*t *= -3.34).

Time-dependent changes in the MODS scores are shown in Figure 2C. No statistically significant difference in MODS scores appeared between survivors and nonsurvivors at day 1. However, serial measurements reveal that although the MODS scores of the nonsurvivors tended to remain at a high level, the MODS scores of the survivors decreased with elapsed time, resulting in a significant overall difference between the two groups of patients (*t *= 8.85).

As reflected by the magnitude of the *t *statistic, the overall difference between the survivors and nonsurvivors over time was the greatest for cfDNA and the smallest for protein C. Thus, if we use the average value (across all available plasma samples for each patient) for cfDNA, MODS, or protein C in the ROC analysis to determine the predictive power of each variable for ICU mortality, we would expect the AUC to be the greatest for cfDNA and the smallest for protein C. As expected, we find that the resulting AUC is 0.96 (95% CI, 0.92 to 1.00) for cfDNA, 0.77 (95% CI, 0.66 to 0.88) for MODS, and 0.64 (95% CI, 0.51 to 0.77) for protein C.

Because serial measurements of cfDNA, MODS, and protein C, averaged across blood samples, tend to be at a lower risk for random and nonrandom errors than the baseline data, we explored the possibility that combining either MODS, or protein C, or both with cfDNA may enhance the predictive power of cfDNA. We found that combining average MODS with average cfDNA increases the AUC from 0.965 to 0.969, whereas combining average protein C with average cfDNA increases the AUC to 0.977. Both of these increases are statistically significant (the *P *values are 0.027 and 0.008 for the coefficient of average MODS and average protein C, respectively). Combining *both *average MODS and average protein C with average cfDNA increases the AUC further to 0.981. However, the coefficient of MODS is not significantly different from zero. It is useful to note that, even though average protein C is weaker than average MODS in predictive power when used alone, the former enhances the predictive power of average cfDNA to a greater extent than does the latter, because a greater overlap in predictive power exists between average MODS and average cfDNA.

An important finding is that the simultaneous use of average cfDNA and average protein C to make predictions of ICU deaths increases sensitivity without sacrificing specificity. The best cut-off point is 2.47 ng/μl for average cfDNA and 0.66 U/ml for average protein C. At this value, sensitivity is increased from 87.9% (95% CI, 77.2 to 99.2) to 97.1% (95% CI, 91.3 to 100), whereas specificity remains at 93.5% (95% CI, 86.2 to 100). The assessment of the average variables in terms of PPV and NPV yields similar inferences (Table 4). With PPV = 0.84 (95% CI, 0.72 to 0.96) and NPV = 0.93 (95% CI, 0.85 to 1.00), the prognostic utility of average cfDNA is greater than those of average MODS and average protein C. The prognostic utility of average cfDNA is enhanced by combining with either average MODS or average protein C. With PPV = 0.92 (95% CI, 0.82 to 1.00) and NPV = 0.98 (95% CI, 0.93 to 1.00), the combination of average cfDNA and average protein C is of the greatest prognostic utility.

**Table 4:** Positive predictive value (PPV) and negative predictive value (NPV) for assessing the prognostic utility of the average cfDNA, average MODS, and average protein C

Variable	Positive predictive value	95% CI	Likelihood ratio χ^2^	Negative predictive value	95% CI	Likelihood ratio χ^2^
cfDNA	0.84	0.72-0.96	18.49	0.93	0.85-1.00	37.85
MODS	0.70	0.54-0.86	5.26	0.77	0.64-0.89	14.01
Protein C	0.74	0.54-0.94	4.44	0.67	0.55-0.79	7.38
cfDNA and MODS	0.91	0.81-1.00	26.84	0.93	0.86-1.00	41.59
cfDNA and protein C	0.92	0.82-1.00	29.25	0.98	0.93-1.00	51.45

Taken together, these studies suggest that cfDNA may predict ICU death better than other predictors examined in this study, and that using the information obtained from repeated measurements, the predictive power of cfDNA can be enhanced by combing it with protein C or MODS in a multivariate statistical model.

### Relation of cfDNA to type of sepsis

To study the relation between cfDNA and causative microorganisms, each case of sepsis was classified in one of five class groupings: Gram-positive bacteria, Gram-negative bacteria, fungal, mixed microbial infections, and unknown or none. The number of patients in each grouping is summarized in Table 1. We observed no statistically significant difference in cfDNA levels among these groupings.

### Source of cfDNA: host versus microbe?

TLR9 is expressed by many cell types in the intestinal tract, including epithelial cells, dendritic cells, and macrophages [26]. The stimulatory ligand for TLR9 is unmethylated cytosine-guanine (CpG) DNA, a microbial product that is recognized by the vertebrate innate immune system. Engagement of TLR9 can elicit an inflammatory response through activation of the Rel/NF-κB transcription factor family and secretion of proinflammatory cytokines [27]. By using 293XL-hTLR9 reporter cells, we determined the levels of NF-κB activation by these cells in response to cfDNA from healthy volunteers (*n *= 5) and from severe sepsis patients (*n *= 18). None of these samples stimulated the production of SEAP from the 293XL-hTLR9 cells, suggesting that the cfDNA from severe sepsis patients is host derived (data not shown).

**Figure 3:**
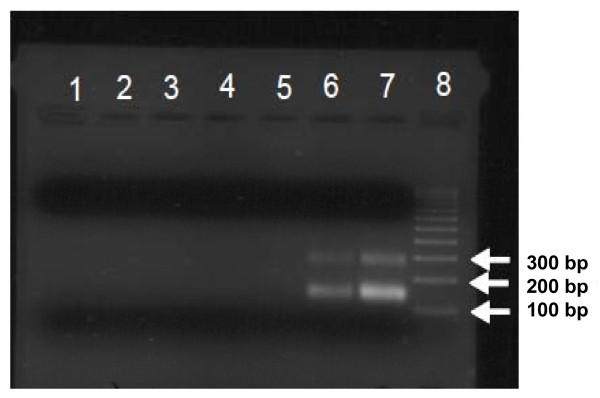
**Agarose gel electrophoresis of cfDNA from severe sepsis patients**. cfDNA was purified from 250 μl of plasma, as described in Materials and methods, and 16 μl was loaded per lane. Lanes 1 to 5, cfDNA from severe sepsis patients (survivors); lanes 6 and 7, cfDNA from severe sepsis patients (nonsurvivors); lane 8, 100-bp DNA ladder.

To visualize the cfDNA from severe sepsis patients, we subjected the DNA to agarose gel electrophoresis. We observed that nine of 80 patients had two prominent DNA bands of ~150 bp and ~300 bp (Figure 3). For the remaining 71 patients, no prominent bands or distinct fragmentation patterns were observed, even after concentrating the DNA 10-fold (data not shown). cfDNA isolated from five healthy volunteers also did not show prominent bands (data now shown). To determine the DNA sequence composition of the two prominent bands, the bands were excised from the gels, inserted into a cloning Vector (Litmus 28i), and sequenced. Table 3 shows sample DNA sequences obtained from these prominent bands. These sequences were identified with nucleotide BLAST (blastn) against human and microbial reference genomic sequences. BLAST results indicated overall closer identity to human genomic sequences (97% to 100% identity) than to microbial sources (Table 5). No human mitochondrial sequences were identified from these two prominent bands, although we acknowledge that the total number of sequences that we obtained from our cloning strategy was small. Taken together, these two sets of experiments indicate that the cfDNA is host derived.

**Table 5:** Sample sequences of cfDNA from severe sepsis patients

Name	Sequence	Source	Chromosome	GenBank Accession Number	Identity
**150-1**	AGAGTCTTGGCATCCATGATAAGTGGGGGTGAGCGGAGGGAAAGACCAAGCCCCAGGACAGCACACTGACCATTCCAGGAGCCAGCATGGGTGGCCCACACACATGGAAGAACTACAGCCCAGACAAGCAGGGCCGCACCAACAGAGGTCCTGCAG	*Homo sapiens*	Chromosome 5 genomic contig, reference assembly	NT_006713.14	100%

**150-2**	GATCCAGACCCTTTCTTTTTTTTTTTTTTTTTTTTTTTTTTGANACAGAN NCTTGCTCTGTCGCCAGGCTGGAGTGCAATGGCGCGATCTCAGCTCACTGCAACCTCCGCCCCCCGCCCCCCGGTTCAAGCGATTCTCCTGCCTCAGCCTCCCACGATCC	*Homo sapiens*	Protein kinase C, α (PRKCA) on chromosome 17	NG_012206.1	97%

**150-3**	CCTGATTTTCCAGGTGCCGTCTGTCACCCCTTTCTTTGACTAGGAAAGGGAACTCCCTGACCCCTTGCGCTTCCTGAGTGAGGCAATGCCTTGCCCTGCTTCGGCTCGCACACGGTGCGTGCACCCACTGACCTGCGCCCACTGTCTGG	*Homo sapiens*	Protein kinase, cAMP-dependent, catalytic, β (PRKACB) gene	DQ_667174.1	100%

**150-5**	CGCGGCGAGGGGGGTAAAAAGCCGCGTTGGCAAAAACCGCGGCGGCGGGGAGCAAAAAGCCGCCGCGGTGGGCGCAAAAAGTCGCCGCGGCCAAAAAGCCGTGCCGGTGGCGGCGGCGGCAAAAAGCCGCGGCGTCGGGGGCGGGG	*Homo sapiens*	Chromosome 21 genomic contig, reference assembly	NT_113952.1	100%

**150-6**	CCCGTAAAGACCCAGGTCACAGGCCACTGTGGCGGAGGGCAGACCCAGAGGCATGGTGACCGGTGCGGGAGAGGGCAGGCCAGCTTCAGGGTGCAGACCCCGCAGAAGCCCGGCTTCACTGGCTCCAGGGTTGTTGCAGGGGGG	*Homo sapiens*	Chromosome 1 genomic contig, reference assembly	NT_004487.18	100%

**150-7**	CCAGGCAGCCAGGGCGCGGCTGAGGTGGGGTGAGGAGGGAGCGCGGGGCGGGCCGTCCGCCTTGCGTGGGAAGCCGCACCCCCTGCAGATGCCGTGGGCGTTTGTCTCTGCCCCCCCCAGGCACCGGCATCGTCAGCCCAGG	*Homo sapiens*	Chromosome 16 genomic contig, reference assembly	NT_037887.4	100%

**150-8**	CCTGTGTGGCCGGAGCCTCCGCGATGAGCACTGCCCCCTGCTCCACGGCACCCAGTCCCATCAACCACCCAAGGGCTGAGGAGTGTGGGCTCATGGCACAGGACTGGCAAGCAGCTCCACCTGCAGCCCCAGTGCAGGATCCACTGGG	*Homo sapiens*	Chromosome 7 genomic contig, reference assembly	NT_007914.14	86%

**300-1**	GACCCATCTGGCCGCCTCCCGAGAGGCCATGGGCGCTGTGACTCCTTCATCTTGGCCTAGGAAGCACCAGCCTTCAGCTGCTCACGCCAGATTCTTGCAGACATTGCAACTCCTCTTTTTCTCGGCTCTACCTTCCACAAACATCCCCTGCTACTGCCAGGACCAGGCCCCGGCCCCGATCCCGGCCCGGTCCACCGCAGCCCATCCCCGCACTGGCTCCTTGCTGCCCCCGACCCTCCCAGCAGCCAGAGGGACTTTTCACC	*Homo sapiens*	Chromosome 9 genomic contig, reference assembly	NT_024000.16	100%

**300-2**	GATCCCCCTCCCAAGAAGCGCCCGGCCCGGGCCGGCCCAGCGGGAGCACCTGAGCTGTTCTGGGCCTCCAGCGTCCTGTGCCCTGCAGAGGCGGGTCCTAGGGAGGCAGAGCTGAGGTGGGGAGGTGGGGGACAGGTCTAGAGAGGATCAGGCACGGCGCGCCCTCGGCCAAGGGCCCCCACCCCAAACATTTCTCCCTGCTGGTCGGCCTTCTCGTTCCCACTCCGCAGGAACCACTGGAAGACAGGCTTCCGGGGAAAACGGCCTGGGGTTTACAAATAACCCAGGTC	*Homo sapiens*	Chromosome 11 genomic contig, alternate assembly	NW_001838025.2	100%

**300-3**	CCACACCTGGATCTGACTGCCCCANNGCCCTTCAGGGCCCTTTGAGGGGGTGATGGGGACAATGTGGAAAGAGGGGGAGGGAAGTTGGGGGGTCCTGCCCACAGCCCCTGCCTGTCTGCACCTCATGTCCCGCACACACACGCTCAGTGCCTGCCCTGAGGAGTGGCAGACCCATTTTACTTTCTTAGAGTAGAGGAGGAAGAGGTGCAGGAGGAAGGCCAGGTAGGAGGGCTGGTAGGGCCAGGGCACTCCCCACCACTGACTGCCCCAGAGGGTGACTTGGG	*Homo sapiens*	Chromosome 17 genomic contig, reference assembly	NT_010718.15	99%

**300-4**	AACCAGGCCTCAGGTGCAGGCCCCACATGACAGATGGACAGACTGAAGTGGGAGGTGGGAGGCGGACACCCCGGCGTCCTGCCAGGAAGGGACACCATCTGCACCTGGCGAGCTGTGGCCTCCAGCCATCGTTTCCCTGCCTAGTTAGGGGCTTTTCCCTCCAGAGCCCTGTCCACTCTGGCCTTGTTTCTGGAACTGCTCCTCACCCGGAGGACCCCATCCTTTCCGTGAAGCAGGCAGTGGGGGCTTTCTGGCAAGTGGCCTCTTCATTAACTATCCCAGAGTGAGTGCAGTC	*Homo sapiens*	Chromosome 16 genomic contig, reference assembly	NT_010498.15	100%

### Potential confounding by renal failure

Because renal impairment is common in severe sepsis, we demonstrated that cfDNA values are not influenced by impaired renal function (that is, no correlation was found between the cfDNA and the glomerular filtration rate (*r *= -0.17)).

## Discussion

DNA can be released from various host cells including neutrophils [28-30], macrophages [31], eosinophils [32], and tumor cells [33]. Neutrophil extracellular traps (NETs), composed of chromatin and granular proteins, not only ensnare bacteria, but also possess neutrophil enzymes that can kill microbes [28]. It has been proposed that NETS and platelet-neutrophil aggregates may hinder the flow of blood in the microcirculation, thereby leading to tissue hypoxia and endothelial damage [29,34]. DNA can also be released by certain strains of bacteria [35,36]. Release of bacterial DNA facilitates the adherence and colonization of bacteria to an inert or living surface [37] and occurs through the release of small membrane vesicles [38].

We demonstrated that the AUC for cfDNA to predict ICU mortality was 0.97 (95% CI, 0.93 to 1.00), suggesting that cfDNA isolated from plasma is a superior discriminator compared with other scoring systems and/or biomarkers [11,25,39,40]. We also studied the time-dependent changes in cfDNA in our sepsis patients. Sequential measurement of cfDNA levels revealed that no overlap occurs in cfDNA levels between survivors and nonsurvivors during the course of the patients' stay in the ICU. This raises the possibility that at a very early stage in severe sepsis, the nonsurviving patients had already reached "a point of no return" given standard clinical management. Thus, in addition to prognostication, levels of cfDNA may be useful in stratifying for inclusion in clinical trials (for example, selecting the most severe cases) or for monitoring response to newer antisepsis therapies or procedures. For example, it is unknown if the current sepsis guidelines for early fluid resuscitation and treatment can modify the production of cfDNA [41].

We also measured temporal changes in protein C and in MODS scores in our cohort of severe sepsis patients. The levels of protein C in our patients are consistent with those published by other studies. For example, we observed that patients who survived exhibited an early directional change in protein C levels to near-normal levels. This finding is consistent with retrospective evaluation of protein C levels in the placebo patients of the PROWESS (Recombinant human activated protein C worldwide evaluation in severe sepsis) trial [42,43]. Indeed, sequential measurements of protein C levels were used to guide administration of rAPC, as reported in the phase II RESPOND trial (Research Evaluating Serial Protein C levels in severe sepsis patients on Drotrecogin alfa [activated]) [44,45]. With respect to serial MODS scores, our data reveal that although the MODS scores of the nonsurvivor patients tended to remain at a high level, the MODS scores of the survivors improved (that is, decreased) over time. This finding is consistent with previous reports that daily MODS scores provide additional prognostic value over baseline MODS [46]. Importantly, our data also suggest that the predictive power of cfDNA can be enhanced by combing it with protein C or MODS in a multivariate statistical model.

To date, only one large study (*n *= 225 patients) examined the prognostic utility of cfDNA in severe sepsis [16]. This study by Saukkonen *et al*. [16] reported that cfDNA concentrations had only moderate discriminative power for ICU mortality (AUC = 0.71 in an ROC curve, 95% CI 0.62 to 0.80), no better than that of clinical scores. In another study of 52 general ICU patients (19 of whom were septic), Rhodes *et al*. reported that the AUC in ROC analysis for baseline cfDNA to predict ICU mortality is 0.84 (95% CI, 0.71 to 0.97) [17]. The major difference between our study and previous reports is the method by which cfDNA levels were quantified. Previous reports quantified cfDNA by using real-time quantitative PCR for the β-globin housekeeping gene, thus detecting a subset of cfDNA; specifically, it would detect nuclear DNA which contains copies of the β-globin gene, but not mitochondrial DNA or DNA from microbial origins. Mitochondrial DNA has been shown to contribute to the inflammatory response in trauma and hemorrhagic shock [47-49]. Furthermore, the PCR-based method for quantifying DNA can be influenced by the fragmentation grade of the DNA in that some degraded β-globin gene copies may not be amplified, resulting in an underestimation of DNA levels. In contrast, because our method of DNA quantification is based on the absorbance at 260 nm, we are able to measure the levels of cfDNA, irrespective of the source or the fragmentation grade of the DNA. By using RT-PCR probing for the β-globin gene, we found that the PCR-based method detected approximately 10-fold less DNA compared with the UV-absorbance method at 280 nm (data not shown).

We observed no difference in cfDNA levels among patients with Gram-positive, Gram-negative, fungal, mixed, or unknown infections. Thus, the release of cfDNA from host cells occurred independent of the infecting organism and hence is likely mediated by the inflammatory mediators generated during the exacerbated host response to infection. We also did not find a relation between cfDNA levels and renal function. These findings are consistent with previous reports that examined the association between nucleosome levels, microorganisms, and renal function in patients with systemic inflammation, sepsis, and septic shock [50]. Nucleosomes are the basic units of DNA packaging in cells, consisting of DNA fragments of approximately 147 bp wound around histone core proteins [51]. Although the sample size was small, the study reported a relation between nucleosome levels and disease severity but not with microorganisms or renal function [50].

Apoptotic cells typically release DNA "ladders" consisting of multiples of 180-bp fragments [52], whereas necrotic cells contain DNA fragments larger than ~10,000 bp [53]. Agarose gel electrophoresis revealed that cfDNA from nine of 80 patients had two prominent DNA bands of ~150 bp and ~300 bp (Figure 3). DNA sequence analyses indicated that the DNA within these bands is of host origin, representative of both noncoding and coding portions of the human genome. We hypothesize that the two prominent DNA bands represent nucleosome units that are resistant to degradation by circulating DNase enzymes. Recent studies showed that extracellular histones are major mediators of death in animal models of sepsis [54] and that histones activate TLR-2 and TLR-4 [55]. In addition, histones promote thrombin generation via platelet-dependent mechanisms and by impairing thrombomodulin-dependent protein C activation [56,57]. Furthermore, we and others have shown that extracellular DNA activates the blood-coagulation cascade [58,59]. Thus, elevations of cfDNA likely reflect a severe pathologic condition whereby DNA and/or histones exert proinflammatory and procoagulant effects.

Limitations to our studies include the following. The sample size is small (*n *= 80), and the stored plasma samples were obtained from severe sepsis patients recruited between 2001 and 2006. The patients were recruited on the basis of convenience because of limitations in funding and research personnel. Thus, bias may have been generated by nonconsecutive patient enrollment. We are currently validating our findings prospectively with consecutive enrollment in a large, multicenter, observational study (ClinicalTrials.gov Identifier: NCT01355042).

## Conclusions

In summary, we identified a biomarker that appears to have high discriminative power to predict ICU mortality in a cohort of patients admitted to the ICU with severe sepsis. These data are also the first to reveal the time-dependent changes in cfDNA levels in patients with severe sepsis. The serial data suggest that the combination of cfDNA with protein C and MODS scores may yield even stronger predictive power. Incorporation of cfDNA in sepsis risk-stratification systems (for example, the PIRO model for staging severe sepsis [60,61]) may be valuable for clinical decision making or for inclusion into sepsis trials.

## Key messages

• cfDNA appears to have high discriminative power to predict ICU mortality in patients with severe sepsis.

• Serial studies suggest that the combination of cfDNA with protein C and MODS scores may yield even stronger predictive power.

• We are currently validating our findings prospectively with consecutive enrollment in a large, multicenter, observational study (ClinicalTrials.gov Identifier: NCT01355042).

• The cfDNA from sepsis patients appears to be host derived.

## Abbreviations

APACHE: Acute physiology and chronic health evaluation; AUC: area under the curve; cfDNA: cell-free DNA; CpG: unmethylated cytosine-guanine; MODS: multiple organ dysfunction score; PC: protein C; ROC curves: receiver operating characteristic curves; SEAP: secreted alkaline phosphatase; TAT: thrombin-antithrombin; TLR: Toll-like receptor.

## Competing interests

The authors declare that they have no competing interests.

## Authors' contributions

DD and LT participated in the study design and helped to collect the data. LS participated in the sequencing and bioinformatic analyses. KL and JP assisted with the statistical analysis. DD, AEF, DJC, JW, and PL conceived of the study, participated in its design and coordination, and helped to draft the manuscript. All authors read and approved the manuscript.
